# Karyotype Reorganization in Wheat–Rye Hybrids Obtained via Unreduced Gametes: Is There a Limit to the Chromosome Number in Triticale?

**DOI:** 10.3390/plants10102052

**Published:** 2021-09-29

**Authors:** Olga G. Silkova, Yulia N. Ivanova, Dina B. Loginova, Lilia A. Solovey, Elena A. Sycheva, Nadezhda I. Dubovets

**Affiliations:** 1 Institute of Cytology and Genetics, SB RAS, Lavrentiev av., 10, 630090 Novosibirsk, Russia; kabanenko@bionet.nsc.ru (Y.N.I.); loginova.dina@gmail.com (D.B.L.); 2 Institute of Genetics and Cytology, National Academy of Sciences of Belarus, Akademicheskaya 27, 220072 Minsk, Belarus; lili_solovei@mail.ru (L.A.S.); E.Sycheva@igs.by (E.A.S.); n.i.dubovets@igc.by (N.I.D.)

**Keywords:** wheat–rye amphidiploids, karyotype reorganization, FISH, C-banding, meiotic restitution, sterility

## Abstract

To date, few data have been accumulated on the contribution of meiotic restitution to the formation of *Triticum aestivum* hybrid karyotypes. In this study, based on FISH and C-banding, karyotype reorganization was observed in three groups of F_5_ wheat–rye hybrids 1R(1A) × R. Aberrations, including aneuploidy, telocentrics, and Robertsonian translocations, were detected in all groups. Some of the Group 1 plants and all of the Group 2 plants only had a 4R4R pair (in addition to 1R1R), which was either added or substituted for its homeolog in ABD subgenomes. In about 82% of meiocytes, 4R4R formed bivalents, which indicates its competitiveness. The rest of the Group 1 plants had 2R and 7R chromosomes in addition to 1R1R. Group 3 retained all their rye chromosomes, with a small aneuploidy on the wheat chromosomes. A feature of the meiosis in the Group 3 plants was asynchronous cell division and omission of the second division. Diploid gametes did not form because of the significant disturbances during gametogenesis. As a result, the frequency of occurrence of the formed dyads was negatively correlated (r = −0.73) with the seed sets. Thus, meiotic restitution in the 8n triticale does not contribute to fertility or increased ploidy in subsequent generations.

## 1. Introduction

Polyploidy plays a central role in plant genome evolution and in the formation of new species [1,2]. In addition to the ancient process of genome-wide duplication in all seed plants, in most plant species, including cultivated ones, two or more divergent genomes can merge via hybridization in a single nucleus [3]. The high heterozygosity of such allopolyploid species ensures the high genetic diversity of their progeny [4,5].

The formation of polyploids is followed by their passing through a bottleneck of instability [6]. When two parental genomes join to form an allopolyploid genome, a “genomic shock” is experienced [7]. A multitude of evolutionary processes affects polyploid genomes, including rapid and substantial genome reorganization, transgressive gene expression alterations, gene fractionation, gene conversion, genome downsizing, and the sub- and neofunctionalization of duplicate genes [2,5,8,9,10,11,12,13,14,15]. Thus, new polyploid species, most of which have experienced several cycles of polyploidization [16], end up suffering a massive loss of “redundant” DNA and the restructuring of their chromosomes, as well as a repeated reduction in genome size [17]. Other changes in genomes at the chromosomal level involve duplications, deletions, fissions, fusions, translocations, and inversions of whole chromosomes, chromosome arms, or smaller segments [18].

The Poaceae family includes many typical allopolyploids. It has been found that the bread wheat subgenomes A, B, and D were originally derived from three diploid (2x; 2*n* = 14) species within the tribe Triticeae: *Triticum urartu* (AA), an extinct or undiscovered species in the lineage of *Aegilops speltoides* (BB), and *Ae. tauschii* (DD) [19,20]. The coadaptation of bread wheat subgenomes is evidenced by their structural and functional dissymmetry, manifesting itself as regional asymmetrical gene distribution and a lack of interaction at the transcriptional level of gene regulation, and by homologous exchange within genes [21,22,23,24,25,26,27,28].

The evolution of cereals has involved variations in chromosome structure and number. Recently, Murat et al. [29] and Pont et al. [21] suggested that Poaceae originated from an Ancestral Grass Karyotype (AGK), which existed 90 million years ago and contained n=7 protochromosomes. In the first step of paleoploidization—paleotetraploidization—a whole-genome duplication (WGD) produced *n* = 14 chromosomes 65 million years ago. This was followed by reciprocal translocations, inversions, and telomeric/centromeric fusions to reach an *n* = 12 chromosome intermediate [29]. Common wheat developed in the same way [25,29]. It is hypothesized that the Ancestral Triticeae Karyotype (ATK) originated from the 12 chromosomes of the AGK intermediate by means of six fusions and one fission (*n* = 7), followed by two rounds of neohexaploidization (involving progenitors/subgenomes A, B, and D) that finally shaped the 21 modern bread wheat chromosomes [21]. Although gross chromosome homologies are conserved, structural changes involving chromosomes 4A, 5A, and 7B are apparently present in all hexaploid and tetraploid wheat in the Emmer group and 1G-4G-6A^t^ +3A^t^ -4A^t^ in the Timopheevii group [30,31,32,33,34,35]. Intra- and interspecific divergence, accompanied by species-specific chromosome rearrangements, have been found in two wheat groups, Emmer and Timopheevii [36]. Although all extant wild wheat have x = 7, comparative cytogenetics highlights considerable chromosomal rearrangements within and among wild diploid and polyploid species [37,38,39].

Artificial polyploidization and remote hybridization are employed in breeding to increase crop yield [40]. Wild relatives are also used when raising allopolyploids in order to expand the genetic diversity of common wheat by the introgression of valuable alleles [41]. Triticale (×Triticosecale Wittmack), the hybrid of wheat and rye, is an allopolyploid species that is evolutionarily younger than durum or common wheat. The earliest naturally emerging wheat–rye hybrids were discovered at the southeastern experimental agricultural station in Saratov in the late 1920s [42,43]. The plants showed intermediate traits and were described by G.K. Meister as a new botanical species, *Triticum Secalotriticum saratoviense* Meister [44]. The first stable amphiploid triticale (Triticosecale Wittmack) is attributed to Rimpau in 1888 [43]. Cytological examinations of the first triticales raised in Russia and Germany showed that their somatic chromosome number was 56 [44], which indicates the combination of four genomes, BBAADDRR. About 68% of repetitive and low-abundance DNA sequences are lost or modified when combined within a single nucleus [45,46,47]. Allopolyploidization in triticale can also be accompanied by rapid variations in retrotransposons, tandem repeats, regulatory units, coding sequences, and promoter sequences [48,49]. The rye parental genome is more prone to changes than the wheat one. After chromosome duplication, triticale genome reorganization occurs slowly, and most changes are confined to the first five generations [46]. The parental genotype affects genomic changes during allopolyploidization [49]. However, the expression of meiotic genes is highly resistant to changes induced by polyploidization [50]. The analysis of a large set of RNA-seq data showed that neither the level of synapsis, the ploidy level, nor the *Ph1* locus affected overall meiotic transcription during the leptotene–zygotene transition stage in wheat–rye hybrids and doubled wheat–rye hybrids (newly synthesized triticale) [50].

The loss of DNA sequences alters the structures of rye and wheat chromosomes in the triticale chromosome set. This reorganization is also characterized by chromosome set instability, that is, the elimination of chromosomes or whole subgenomes [51,52,53,54,55,56,57,58,59].

Nearly all molecular and cytogenetical studies of genome reorganization have been conducted with triticale raised via colchicination. However, most natural polyploids arose sexually through the formation of unreduced gametes with somatic (2n) rather than haploid (n) chromosome numbers [3,60,61,62]. Polyploids may arise in one step via the fusion of two unreduced gametes or through a so-called triploid bridge. The triploid bridge mechanism seems to occur more often than the single-step pathway because of the low probability of the fusion of two unreduced gametes in natural populations [63]. Functional gametes in wheat–rye F_1_ hybrids are formed via meiotic restitution, in which there is no pairing of chromosomes, and univalents are divided into sister chromatids in the first meiosis after which the meiosis ends [64,65,66]. It was found that meiotic restitution is genetically controlled in wheat–rye hybrids [64,65,66,67,68] and inherited in durum wheat–rye hybrids [54,66,67].

At present, little is known about the possibility of inheriting meiotic restitution, or about the contribution of meiotic restitution to the patterns of karyotype formation and the rate of meiotic stability restoration in common wheat–rye hybrids. Previously, we examined the chromosome sets, structures, and behavior in meiosis of the selected progenies (with good seed-setting ability) of F_2-3_ wheat–rye hybrids, obtained using the bread wheat cv. Saratovskaya 29 and the wheat–rye substitution line 1Rv(1A), which determines meiotic restitution [64]. Karyotype analysis of the F_2_ *Triticum aestivum* L. cv. Saratovskaya 29 × *Secale cereale* L. var. Onochoyskaya (S29 × R) hybrid revealed 56 chromosomes; among them were 42 wheat chromosomes and 14 rye chromosomes [69]. The karyotype of the F_2_ 1Rv(1A) × R hybrid contained 46 chromosomes, of which three pairs of rye chromosomes 1R1R4R4R2RL2RL, 1R1R replaced the chromosomes 1A1A, and 2RL2RL and 4R4R were added. In the F_3_ generation of S29 × R hybrids, the octoploid number of chromosomes with aneuploidy of single rye and wheat chromosomes was preserved, while in the 1Rv(1A) × R hybrids, the number of chromosomes varied from 42 to 49, but in most plants, 2*n* = 46 was retained. The main meiotic disorders in hybrids F_3_ 1Rv(1A) × R and S29 × R was the presence of univalents in the first division and micronuclei in the second. Most disturbances are terminated by the fifth generation of allopolyploid hybrids [46,70]. Based on this, in the current work, we examined the chromosome sets, structures, and behaviors in meiosis of three groups of F_5_ wheat–rye hybrids. Each group is the progeny of a single F_1_ plant obtained by crossing the 1Rv(1A) common wheat disomic substitution line with the rye *Secale cereale* var. Onochoyskaya. The significant elimination of rye chromosomes was observed in the first two groups. The chromosome numbers in Group 3 varied from 52 to 56. All rye chromosomes were preserved there, but the wheat chromosomes showed insignificant aneuploidy. Our observations suggest that genome reorganization is not finished in any group of F_5_ descendants. The meiosis in the hybrids was unstable. Specific features of meiosis in the plants of the third group included asynchronous cell division and the omission of the second division, followed by significant disturbances during mitosis in gametogenesis. In the other two groups, the second division of meiosis took place. Therefore, meiotic restitution in 8n triticale was inherited but did not contribute to increases in ploidy in subsequent generations.

## 2. Materials and Methods

Three groups of F5 plants were obtained by crossing the 1Rv(1A) disomic wheat–rye substitution line (2*n* = 42) (*T. aestivum* L. cv. Saratovskaya 29/*S. cereale* L. cv. Vyatka) [71] with spring rye *Secale cereale* L. var. Onokhoiskaya was investigated. Rye Onokhoiskaya tolerates spring frosts and May–June drought and is resistant to diseases and pests. The 1Rv(1A) disomic wheat–rye substitution line is cytogenetically stable [71] and determines meiotic restitution [64]. The partially fertile F_1_ hybrids 4-3, 4-7, and 73-1 arose from unreduced gametes [64].

**Figure 1:**
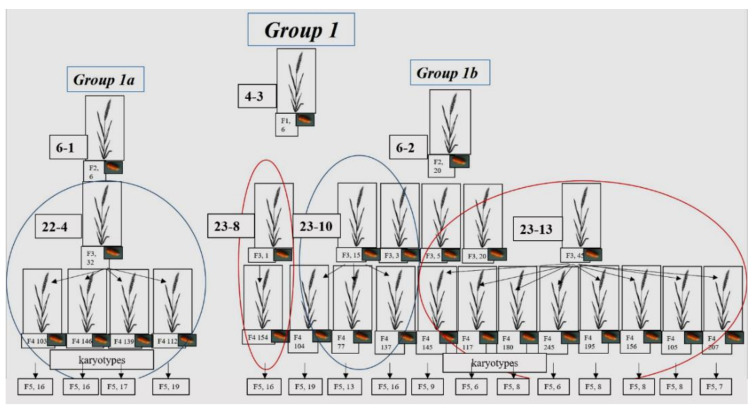
Development of wheat–rye hybrids of Group 1. The scheme shows individual plants from the F_1_–F_4_ generations, and the number of seeds in these plants. For the F_5_ generation, the number of plants analyzed for karyotype is shown.

Six seeds were set in the F_1_ 4-3 plant, and they produced only 2 fertile F_2_ plants (6-1 and 6-2), which were taken for further study (Table S1, Figure 1). As the seeds of each plant were sown separately, starting from F_1_, their progeny was designated as lines. In the F_3_ of plant 6-1 (subgroup 1a), only 1 plant (22-4) was fertile, while 4 high-yield plants were obtained in F_4_. One low-yield plant (23-8) and 2 plants with different numbers of grains (23-10 and 23-13) were chosen from the F_3_ progeny of plant 6-2 (subgroup 1b). A total of 12 high-yield plants were chosen from F_4_ in subgroup 1b (Figure 1). As a result, the chromosome sets were analyzed in the F_5_ plants that originated from 16 F_4_ plants. Some of the grains of one plant were sown in the greenhouse; karyotypes were analyzed in vegetative plants using FISH. Other grains from the same plant were transferred for karyotype analysis using C-banding.

**Figure 2:**
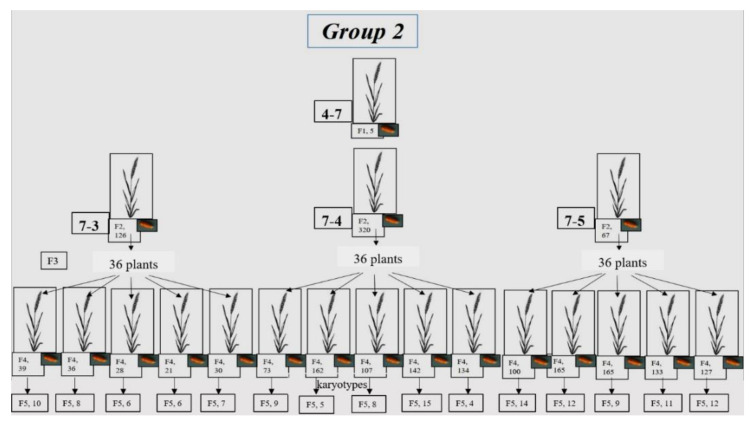
Development of wheat–rye hybrids of Group 2. The scheme shows individual plants of the F_1_–F_4_ generations, and the number of seeds in these plants. In the F_5_ generation, the number of plants analyzed for karyotype is shown.

Five seeds were set in plant F_1_ 4-7 (Group 2). They yielded 5 plants, and 3 high-yield plants were chosen for further crosses. To obtain generation F_3_, 36 seeds were taken from each plant, and 5 high-yield plants from these 36 were selected to obtain F_4_ (Table S1, Figure 2). Finally, chromosome sets were analyzed in F_5_ plants originating from 15 F_4_ plants via C-banding.

**Figure 3:**
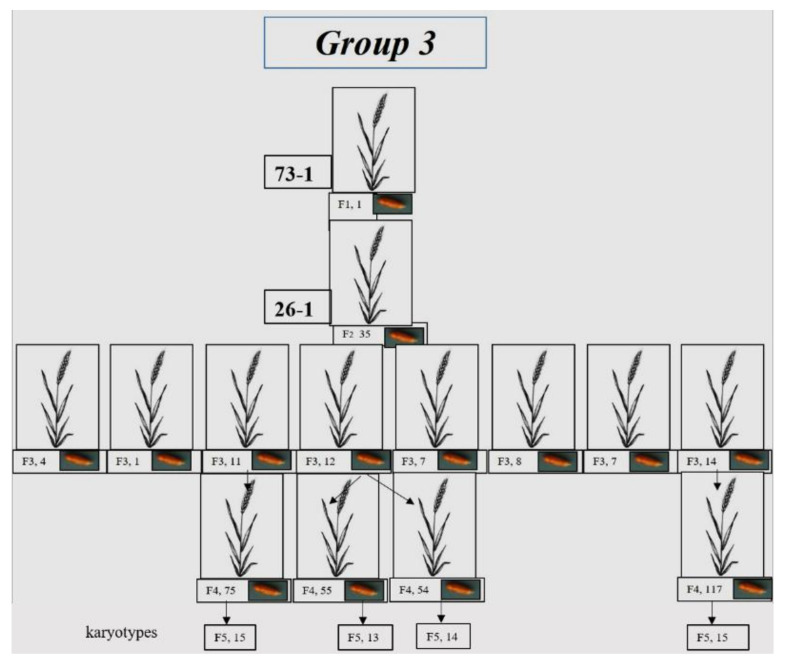
Development of wheat–rye hybrids of Group 3. The scheme shows individual plants of the F_1_–F_4_ generations and the number of seeds in these plants. The number of plants in the F_5_ generation in which the karyotype was analyzed is shown.

One seed was set in plant F_1_ 73-1 (Table S1, Group 3), and 35 seeds in F_2_ (26-1). Generation F_3_ plants were grown from these seeds, only 8 of which were fertile (Figure 3). The 3 plants with the most seeds were selected. In total, 4 plants with the most seeds were chosen from F_4_. Some of the grains of each of the 4 plants were sown in the greenhouse; karyotypes were analyzed in the vegetative plants using FISH. Other grains from the same plants were transferred for karyotype analysis using C-banding.

F_5_ hybrids were grown in a greenhouse under a 24/18 °C day/night temperature regime and a 16/8 h day/night schedule.

### 2.1. Routine Meiosis Analysis

To analyze meiotic division, young spikes were fixed in ethyl alcohol–acetic acid 3:1 and stored at 4 °C. Pollen mother cells (PMCs) were stained with and squashed in 3% acetocarmine. All of the anthers with PMCs at metaphase I–anaphase I and anaphase II–telophase II, and with separate microspores, were examined (Table 1). Each anther was analyzed individually, assaying all PMCs in each anther.

### 2.2. Giemsa C-banding

C-banding was carried out as in [72]. The slides were examined under an Amplival microscope (Carl Zeiss Jena). Images were recorded with a LeicaDS300 camera (Leica Microsystems) and processed using the Adobe Photoshop CC2017 software.

Chromosomes were identified using a generalized species idiogram [73,74].

### 2.3. Fluorescence In Situ Hybridization (FISH)

The mitotic and meiotic slides used for FISH were prepared as in [64]. Meiocytes were analyzed at metaphase I (MI) (Table 1). The following probes were used: *Aegilops tauschii* pAet6-09, specific to centromeric repeats on chromosomes of rice, wheat, rye, and barley [75,76]; pAWRc, specific to the rye chromosome centromeric repeat [77]; rye genomic DNA. The samples of plasmid DNA containing the corresponding repeats were kindly provided by Dr. A. Lukaszewski (University of California, United States). Centromere-specific probe pAet6-09 was labeled with biotin-16-dUTP and pAWRc with digoxigenin-11-dUTP via polymerase chain reaction (PCR). Total rye DNA was labeled by nick translation using digoxigenin-11-dUTP. The probes were used separately or in combination (rye DNA/centromere and pAet6-09/ pAWRc) and were mixed with blocking (sonicated) wheat DNA. Biotinylated probes were detected using avidin conjugated to fluorescein (Fluorescein Avidin D, Vector Laboratories, No. A-2001), and the hybridization signal was amplified using fluorescein anti-avidin (Fluorescein Anti-Avidin D, Vector Laboratories, No. SP-2040). Digoxigenin-labeled probes were detected using anti-digoxigenin antibodies conjugated with rhodamine (Anti-digoxigenin-rhodamine, Fab fragments, Sigma-Aldrich, no. 11207750910 ROCHE). Chromatin was stained with 1 mg/mL DAPI (4′,6-diamidino-2-phenylindole) in Vectashield anti-fade solution (Vector Laboratories).

All slides were examined under an Axio Imager M1 (Carl Zeiss) microscope. Images were recorded with a ProgRes MF camera (Meta Systems, Jenoptic) and processed using the Adobe Photoshop CS2 software.

### 2.4. Statistical Analysis

Associations between 2 traits (the number of grains and the percentage of micronuclei at telophase II, the number of grains, and the percentage of dyads at telophase II) were determined using the Pearson correlation coefficient (Microsoft Excel program). The significance of the correlation was determined using the Student’s *t*-test and the Chaddock scale (Table 2).

## 3. Results

### 3.1. Karyotyping

#### 3.1.1. Group 1

**Figure 4:**
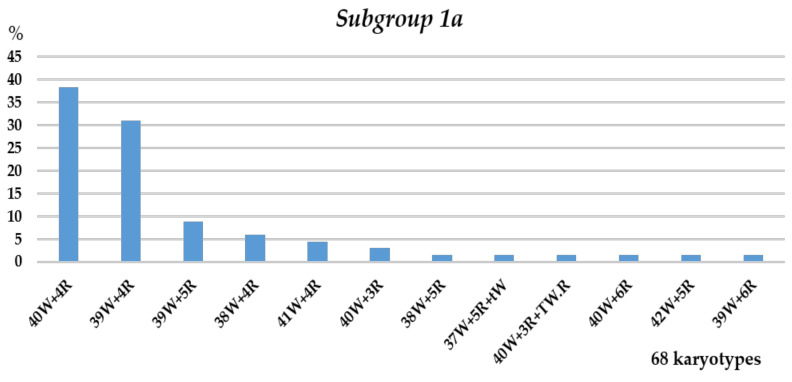
Frequencies of different chromosome sets in the plant karyotypes of subgroup 1a, 22-4 plant progeny. Designations: T, Robertsonian translocation; t, telocentric; W, wheat chromosomes; R, rye chromosomes.

**Figure 5:**
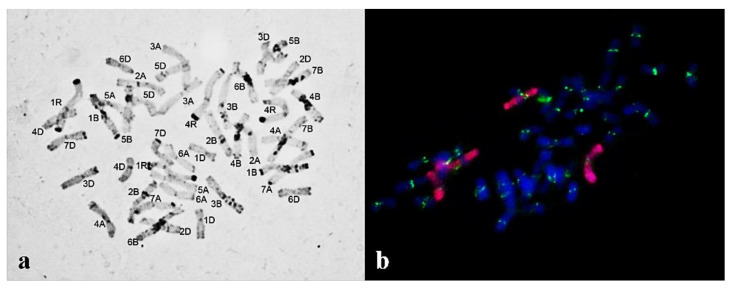
Chromosome sets of plants of subgroup 1a: (**a**) 2*n* = 44, 1R(1A) substitution, 4R4R added (C-banding); (**b**) 2*n* = 44, four rye chromosomes (GISH; rye chromosomes are labeled red and centromeres, green).

The FISH and C-banding data indicate that the chromosome sets of the F_5_ descendants of two sister lines (subgroups 1a and 1b) differ. Plants with 2*n* = 44 and 2*n* = 43 (47.05 and 36.76%, respectively) were predominant in subgroup 1a (Figure 4). The numbers of rye chromosomes varied from three to six, and most sets (79.4%) displayed four rye chromosomes, namely, disomic 1R and 4R (Figure 5). Chromosome 1R was trisomic or tetrasomic in sets with six rye chromosomes. Wheat chromosomes were mostly present in the disomic state, and chromosomes 3B, 4A, 4D, 5D, and 7D were monosomic in sets one, two, one, one, and one, respectively. The rye chromosome 1R always replaced wheat 1A. Chromosome 4R replaced chromosome 4D in one plant and 4A in two. In other plants, this chromosome was added to the wheat chromosomes.

**Figure 6:**
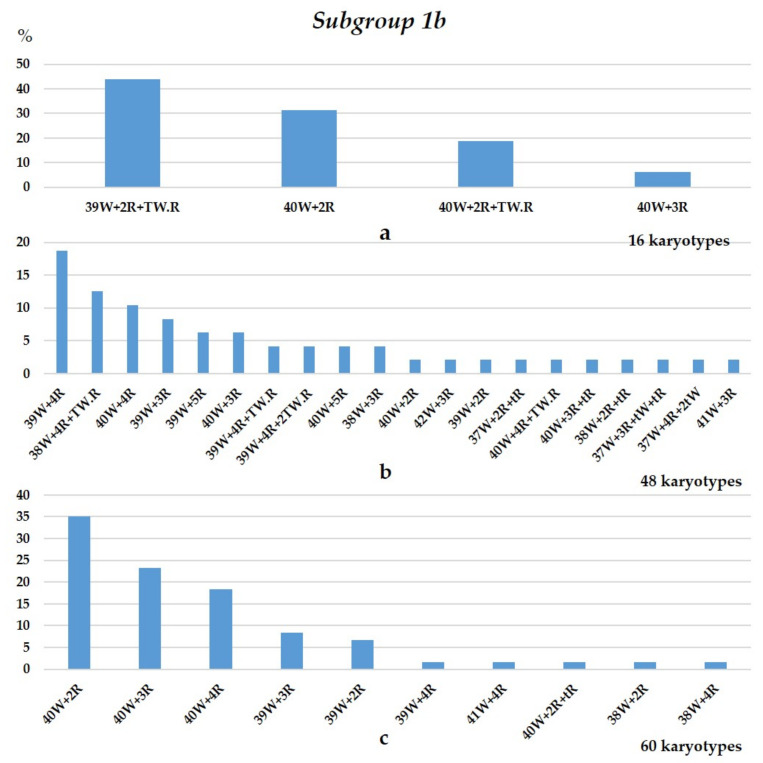
Frequencies of different chromosome sets in the plant karyotypes of subgroup 1b: (**a**) 23-8 plant progeny; (**b**) 23-10 progeny; (**c**) 23-13 progeny. Designations follow Figure 4.

Subgroup 1b included chromosome sets of F_5_ plants obtained from three F_3_ plants (Figure 1 and Figure 6). This subgroup was distinguished by the presence of the three rye chromosomes 1R, 2R, and 7R, while 4R was absent. Chromosome 1R was found in the disomic state in all plants, and it replaced wheat chromosome 1A. The chromosomes 2R and 7R were monosomic. This group was also marked by wheat–rye Robertsonian translocations and wheat and rye telocentric chromosomes (Figure 6).

**Figure 7:**
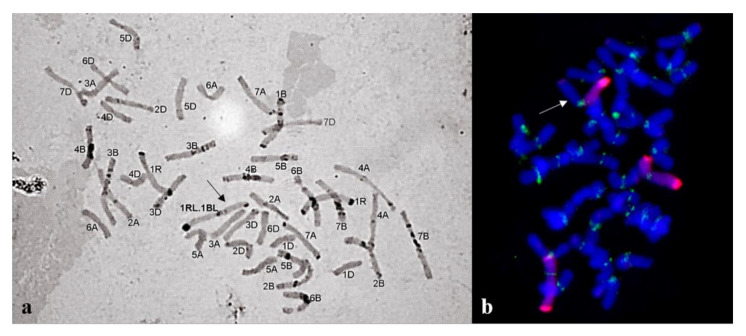
Chromosome sets of the same plant progeny of subgroup 1b with a 1BL.1RL translocation (2*n* = 42) (shown with an arrow): (**a**) C-banding; (**b**) GISH; rye chromosomes are labeled red and centromeres with green.

Chromosome sets in the progeny of plant 23-8 (Figure 7a) formed three major groups: 39W+1R1R+T1BL.1RL (43.75%) (Figure 6a), 40W+1R1R (31.25%), and 40W+2R+T1BL.1RL (18.75%). The three major groups in the progeny of 23-13 were 40W+1R1R (35%), 40W+1R1R+2R (23.33%), and 40W+1R1R+2R+7R (18.33%) (Figure 6c). Wheat chromosomes were in the disomic state with few exceptions (3A, 2D, and 4B were monosomic).

**Figure 8:**
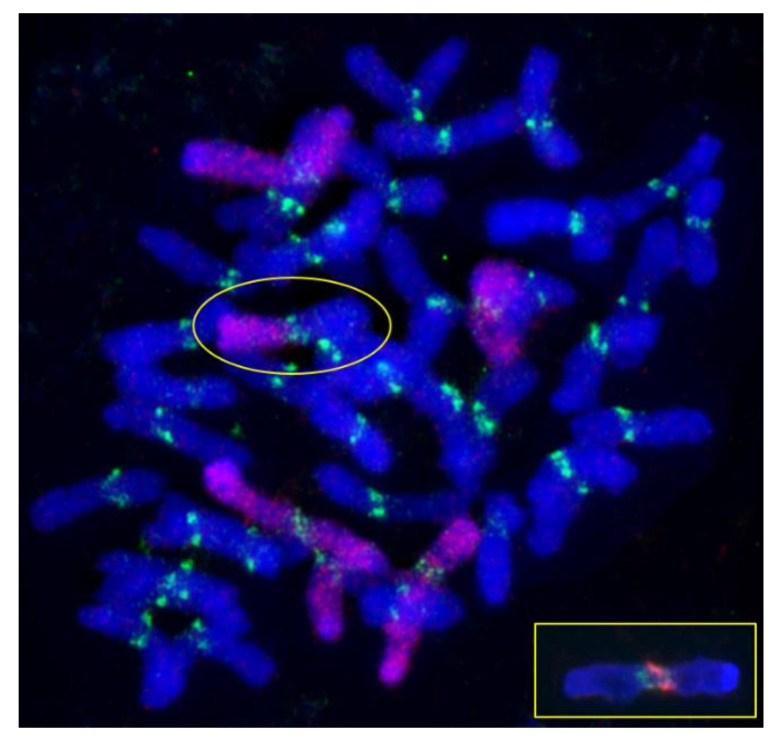
A chromosome set with a wheat–rye Robertsonian translocation (in circle). GISH, rye chromosomes are labeled red and pAet6-09, green. The inset shows the chromosome centromere with two non-overlapping probes pAet6-09 (green) and pAWRc (red).

A different pattern was observed in the progeny of plant 23-10. Their chromosome sets were more diverse: a total of 20 different sets were found (Figure 6b). The most frequent chromosome sets were 39W+1R1R+2R+7R (18.75%), 38W+1R1R+2R2R+T2RL.W (12.5%), and 40W+1R1R+2R+7R (10.41%). The numbers of rye chromosomes varied from two to five. The 40W+1R1R set was found in only one plant. Sets with wheat and rye telocentrics and with Robertsonian translocations were found in 33.33% of plants. The translocated chromosomes had hybrid centromeres because the centromeric repeats pAet6-09 and pAWRc did not overlap (Figure 8).

#### 3.1.2. Group 2

**Figure 9:**
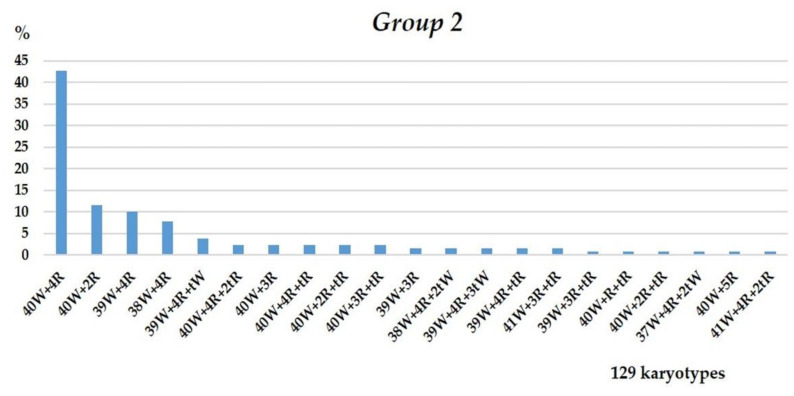
Frequencies of different chromosome sets in plant karyotypes of Group 2. Designations follow Figure 4.

**Figure 10:**
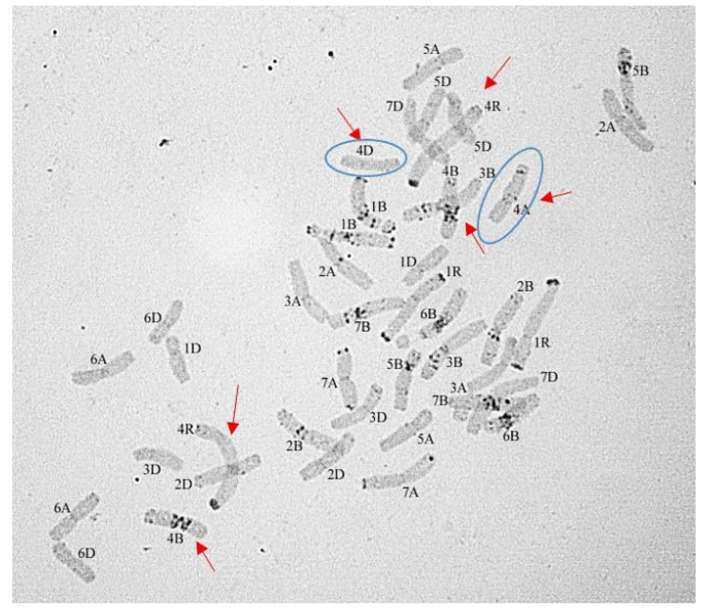
Karyotype with disomic substitution 1R(1A) and monosomic substitutions 4R(4A) and 4R(4D) 2*n* = 42. C-banding.

The chromosome sets of the F_5_ plants in Group 2 were relatively uniform. As shown by C-banding, only two rye chromosomes were present in the disomic state: 1R1R and 4R4R (Figure 9 and Figure 10).

The intergenomic substitution 1R(1A) was preserved in the first homeologous group in all sets. A 4R4R pair was added to the whole set of common wheat chromosomes in 58.59% of sets. Alternatively, it replaced one of three wheat chromosomes of the fourth homeologous group in 13.15% of sets. Monosomic was observed in 11 chromosome sets (Figure 10), and disomic substitution in 6: in 1 plant with 4R(4A) substitution and in 5 with 4R(4B) (Table 3). Chromosome 4B was the most commonly eliminated or rearranged (19 plants, Table 3). Aneuploidy for chromosomes 6A, 5B, and 7D was detected in 11 plants—7, 1, and 3, respectively.

#### 3.1.3. Group 3

**Figure 11:**
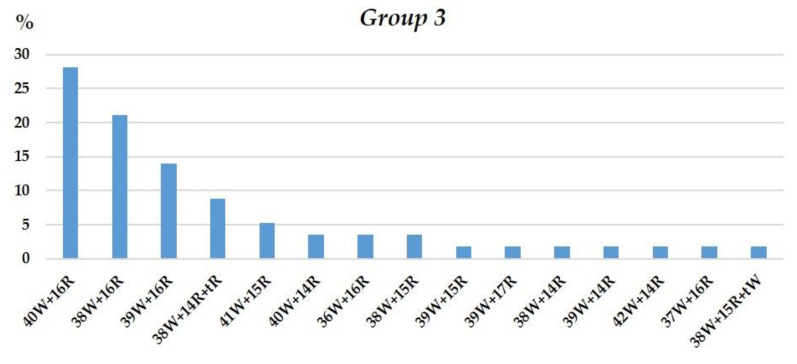
Frequencies of different chromosome sets in plant karyotypes of Group 3. Designations follow Figure 4.

The chromosome numbers in Group 3 plants were nearly octoploid, varying from 52 to 56. Plants with 2*n* = 56 constituted 36.84%; 52, 14.03%; 53, 8.77%; 54, 26.3%, and 55, 14.03% (Figure 11).

**Figure 12:**
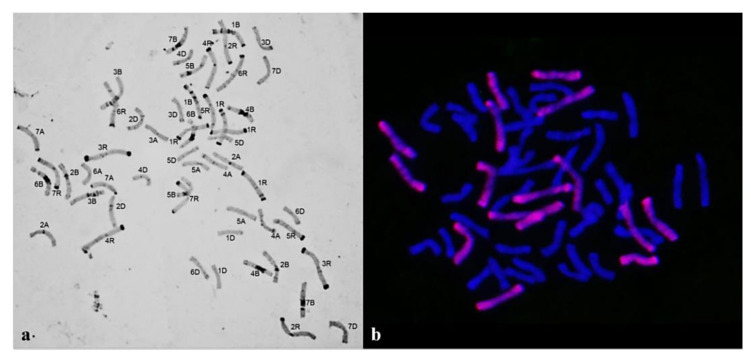
Chromosome sets of plants of Group 3: (**a**) Chromosome number 2*n* = 53, 1R(1A) substitution, and monosomic substitutions 3R(3A) and 6R(6A), C-banding; (**b**) 2*n* = 56, 40W+16R; GISH. Rye chromosomes are labeled red.

The presence of 16 rye chromosomes owing to chromosome 1R tetrasomy (68.4% of plants) was a specific feature of the chromosome sets (Figure 11). The 40W+16R chromosome combination was found in 28.07% of the sets. Four sets lacked one pair of 1R chromosomes, and one set lacked one 6R chromosome. Intergenomic substitution 1R(1A) was preserved in homeologous group 1. It was found in all chromosome sets but four. Disomic and monosomic intergenomic substitutions of wheat chromosomes 3R(3A), 6R(6A), 2R(2B), 3R(3D), and 4R(4D) were detected in nine plants, of which six showed substitutions of the chromosomes of homeologous group 3 (Figure 12). Chromosomes with altered structures were identified by GISH in only six plants—five with a rye telocentric and one with a wheat telocentric.

### 3.2. Chromosome Behavior in Meiosis

**Figure 13:**
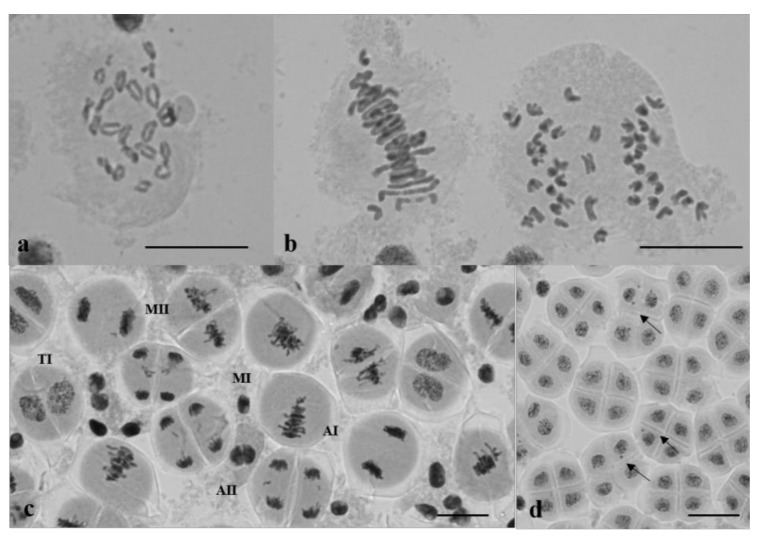
The behavior of chromosomes undergoing meiosis in plants of Group 1: (**a**) Diakinesis—chromosomes form bivalents; (**b**) metaphase I and anaphase I. Two univalents in a meiocyte at metaphase I; univalents arrested on the metaphase plate at anaphase I; (**c**) cell cycle asynchronization in meiocytes in one anther; meiocytes at MI–AII; (**d**) meiocytes at TII; micronuclei are shown with arrows. Staining acetocarmine. Scale bar 10 μm.

Chromosome behavior was studied in plants from Groups 1 and 3. The predominant meiotic aberrations in Group 1 were (i) the formation of univalents and their improper disjunction, leading to the formation of micronuclei, and (ii) cell cycle asynchronization (Figure 13).

Meiocytes with univalents constituted 68.3 ± 2.64% to 100% of subgroup 1a and 60 ± 3.05 to 92 ± 2.16% of subgroup 1b (progeny of plant 23-10), and made up 32.4 ± 1.05% of the progeny of plant 23-8. Univalents were lagged on the metaphase plate at anaphases I and II. The arrest of chromosomes on the metaphase plate at AII caused the formation of micronuclei at the tetrad stage. The counting of micronuclei in tetrads revealed differences within Group 1 and within each of its subgroups. In subgroup 1a, meiocytes with micronuclei constituted 47 ± 3.75 to 70.26 ± 6.2% (Table 4).

The asynchronous cell cycle manifested itself as the presence of meiocytes from metaphase I to telophase II inside the same anther (Figure 13). Such anthers were noted in all plants, but their frequencies varied broadly, from 13.2 to 64%. The frequency of anthers with asynchronous meiocyte division did not correlate with poor seed sets (r = 0.19).

**Figure 14:**
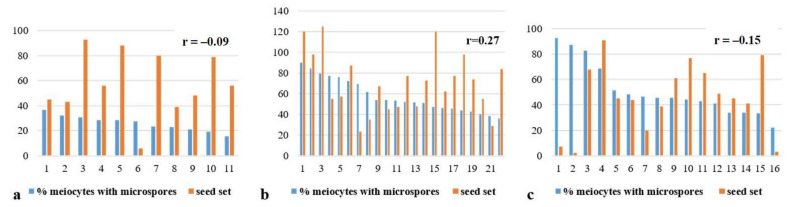
Absence of correlation between the frequency of micronuclei in meiosis and the seed set: (**a)** Subgroup 1a, 22-4 plant progeny; (**b**) subgroup 1b, 23-8 plant progeny; (**c**) subgroup 1b, 23-10 plant progeny. r—correlation coefficient.

In subgroup 1b, the lowest number of meiocytes with micronuclei was found in the progeny of plant 23-8: 13.93 ± 1.89%. In the progeny of 23-10, the percentage of meiocytes with micronuclei varied from 46.5 ± 2.45 to 65.21 ± 9.4%. Seed sets varied among plants within subgroups, and showed negligible correlations with micronucleus numbers, according to the Chaddock scale (Table 1, Figure 14).

**Figure 15:**
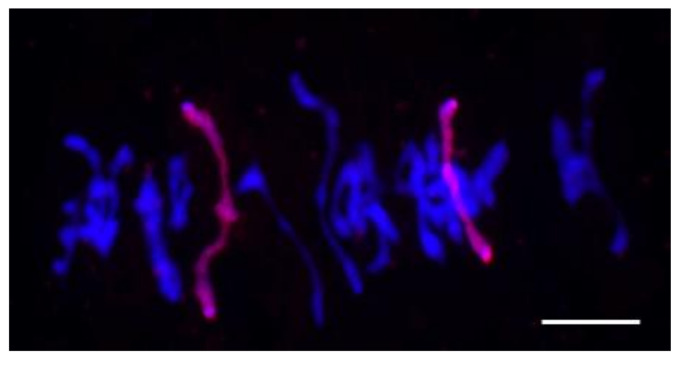
Rye chromosomes 1R1R and 4R4R form rod bivalents at metaphase I. Scale bar = 40 μm.

The rye chromosomes 1R1R and 4R4R were identified in the chromosome sets of subgroup 1a. To understand the cause of the preservation of chromosomes 4R4R up to generation F_5_, we analyzed the behavior of rye chromosomes at metaphase I and found that chromosomes 1R1R and 4R4R together formed 1.82 ± 0.05 rod and ring bivalents per meiocyte (Figure 15). Univalent rye chromosomes were detected in 17.8% of cells.

**Figure 16:**
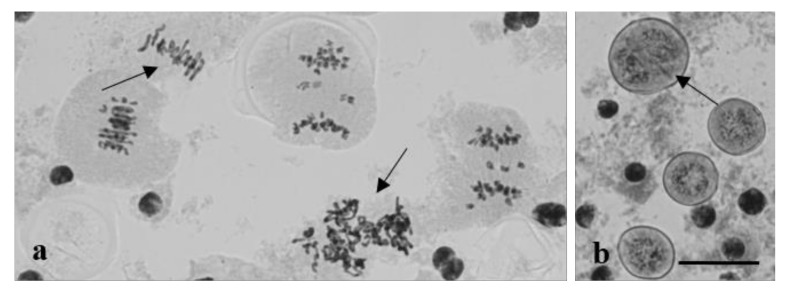
Cytomixis in meiosis in plants of Group 1: (**a**) Chromosomes in the donor cell behave normally, whereas abnormal chromosome behavior is observed in the recipient cell (shown with an arrow); (**b**) pollen grains of different sizes. Staining acetocarmine. Scale bar = 10μm.

Chromatin migration was also detected in meiosis. Cytomixis was identified in MI meiocytes and in pollen grains (Figure 16). Chromosome disjunction was not disturbed in donor cells (with fewer chromosomes). In contrast, the division machinery was inoperative in the recipient cells, and chromosomes occurred in indistinct clusters.

**Figure 17:**
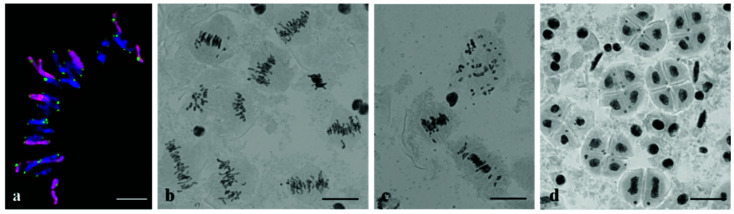
Typical chromosome behavior in meiosis in plants of Group 3: (**a**) Metaphase I; (**b**) metaphase I and anaphase I; (**c**) meiocyte without chromosome pairing; (**d**) tetrads with micronuclei and a dyad amid tetrads. (**a**) GISH; rye chromosomes are labeled red and pAet6-09, green; (**b**–**d**) acetocarmine. Scale bars = 40 μm in (**a**) and 10 μm in (**b**–**d**).

Univalents were found in 77.07 ± 2.01% of the meiocytes in metaphase I in Group 3 plants. They were formed from both wheat and rye chromosomes (Figure 17a,b). Micronuclei were detected in 68.9 ± 3.86% of the microsporocytes in telophase II (Figure 17d). Some meiocytes showed no chromosome pairing at all (Figure 17c).

**Figure 18:**
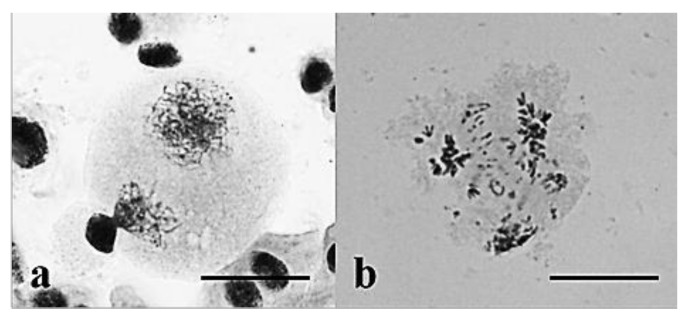
Meiotic aberrations in plants of Group 3. (**a**) Cytomixis; chromatin migrates between a tapetum cell and a meiocyte at prophase I. (**b**) Chromosome breaks at AI. Staining acetocarmine. Scale bar = 10 μm.

Some cells displayed chromatin breakage and cytomixis (Figure 18). The migration of chromatin between a tapetum cell and a meiocyte was detected at prophase I, at which point its compaction changed.

**Figure 19:**
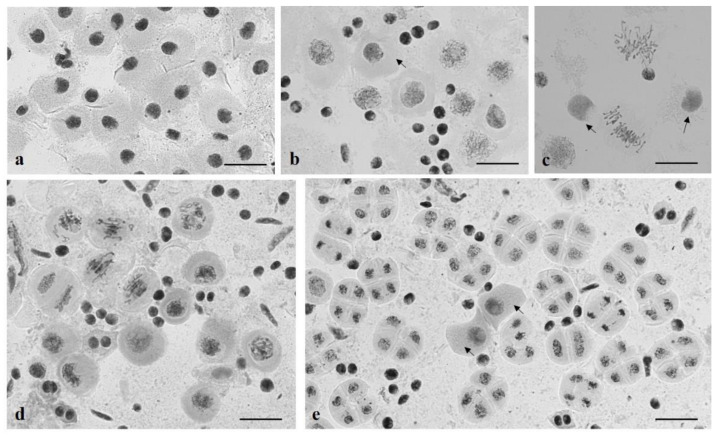
Meiocytes at early prophase stages retard in development in plants of Group 3. (**a**) The leptotene–zygotene stage; all meiocytes develop synchronously; (**b**) a meiocyte at the zygotene stage amid meiocytes at the pachytene stage; (**c,d**) meiocytes at prophase amid meiocytes at MI, TI; **(e**) a meiocyte at the zygotene stage amid meiocytes at the tetrad stage. Staining acetocarmine. Scale bar = 10 μm.

Asynchronous cell cycles were characteristic of meiosis in hybrids of this group. At the leptotene–zygotene stage, all anther meiocytes corresponded to this state (Figure 19a), whereas meiocytes corresponding to leptotene–zygotene was present in all subsequent meiotic phases, from pachytene to telophase II (Figure 19b–f).

**Figure 20:**
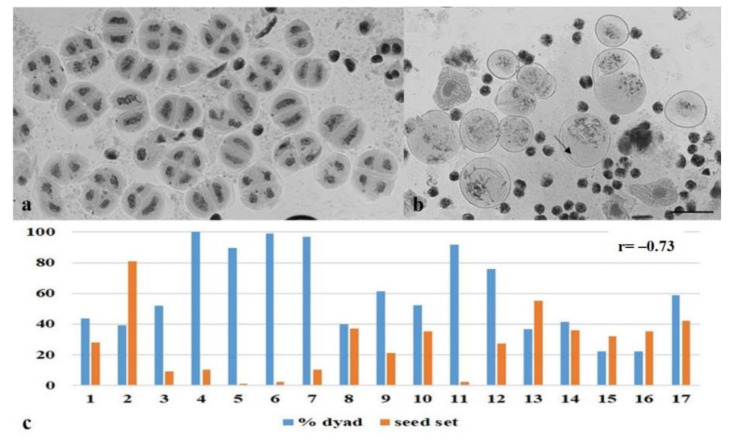
Dyad formation in plants of Group 3. (**a**) Dyads amid tetrads; (**b**) abnormal pollen grain development; meiocytes at the leptotene stage amid pollen grains (shown with an arrow); (**c**) The frequency of dyad formation inversely correlates (r = −0.73) with seed set. r—correlation coefficient. Staining acetocarmine. Scale bar = 10 μm.

The omission of meiotic division II was observed in some plants, and dyads were identified among tetrads (Figure 20a). The percentage of dyads varied from 21.9 to 100 (Figure 20c). Significant aberrations in the mitotic division and chromatin structure were observed during pollen grain formation (Figure 20b).

To summarize, the data on meiosis in particular plants and their fertility show that the high frequency of dyad formation is negatively correlated (r = −0.73) with the seed set (Figure 20c). According to the Chaddock scale, a correlation coefficient r = −0.73 indicates high correlations with marked associations. Dyad formation and seed set were significantly associated (Student’s *t*-test, *p* ≤ 0.001, df = 15).

## 4. Discussion

### 4.1. Chromosome Instability in F_5_ 1Rv(1A) × R Hybrids

Meiotic restitution (spontaneous chromosome duplication in gametes, yielding 2n gametes) in F_1_ of interspecies and intergeneric hybrids is the means by which new polyploids (allopolyploids) arise in angiosperms [78,79,80]. The earliest wheat–rye hybrids, octoploid triticales, were also obtained by means of spontaneous chromosome duplication, including anthers as well as ovules [47]. Although triticales have a complete chromosome set, almost all newly formed triticales produce some chromosomally variable progeny [47,49,65,81,82]. In this study, the F_1_ hybrids from which F_5_ were obtained via self-pollination were produced using unreduced gametes. The unreduction occurs as follows: univalents congregate on the metaphase plate and separate into sister chromatids at AI. Then, two daughter nuclei are formed, and meiosis ends after meiosis I [64]. It was expected that the fusion of 2n gametes would give rise to octoploid triticales. However, the analysis of the chromosome sets of the three groups studied revealed their different means of reorganization. A common feature of the chromosome sets of the three groups was the predominant transmission of chromosome 1R, which replaces wheat chromosome 1A. This was predictable, as line 1Rv(1A) was used for hybridization to rye. One-third of Group 3 plants were octoploids (2*n* = 56) bearing the tetrasome rye chromosome 1R. Other chromosome sets showed aneuploidy for two rye chromosomes and five intergenomic substitutions for wheat chromosomes. In contrast, the chromosome sets in Groups 1 and 2 reverted to the ancestral substitution line 1Rv(1A). They retained 37 to 40 wheat chromosomes and eliminated four to five rye chromosome pairs. The F_2_ hybrid 7-4, the ancestor of the F_5_ of Group 2, comprised a set of 46 chromosomes, with 40 wheat and 6 rye chromosomes (1R1R4R4R2RL2RL), as shown by C-banding [69]. The long arms 2RL2RL were preserved in the F_3_ generation [69] and were eliminated by F_5_. One reason for the absence of rye chromosomes from F_2_ might be their elimination in hybrid embryogenesis [69]. Alien chromosome elimination has been reported in crosses of wheat with *Secale cereale*, species of genus *Hordeum*, and species more distantly related to wheat, such as maize (*Zea mays*), pearl millet (*Pennisetum glaucum*), sorghum (*Sorghum bicolor*), and *Imperata cylindrica* [83,84,85,86,87,88,89,90,91,92,93,94,95,96,97]. The preferred elimination of wheat D-genome chromosomes in the first generations after synthetic wheat (BBAADD) × rye (RR) hybridization was also observed, and an F_2_ seedling carrying 48 chromosomes was observed [56]. Hexaploid triticales with 28 intact A/B and 14 intact R chromosomes, and with other chromosome constitutions, including monosomic, substitution, and translocation lines, were found in F_5_ of these hybrids [54,56]. Another reason for the absence of rye chromosomes from F_2_ may be meiotic irregularities in F_1_, which produce laggard chromosomes and aneuploid gametes [98]. Alterations in chromosome disjunction in wheat–rye hybrids may produce gametes with chromosome numbers other than 21 or 28 [65,81]. The analysis of F_2_ *T. aestivum* L. × *S. cereale* L. indicated chromosome number variability. Plants in one group had the euploid chromosome number 2*n* = 56, and others had aneuploid numbers 49 to 54 [81]. The same study showed that most of megaspores in F_1_ hybrids were aneuploid for one to four chromosomes, wheat or rye. Aneuploid plants were detected among hybrids *T. turgidum* L. × *S. cereale* L. using the meiotic restitution pathway: one was monosomic (41 chromosomes) for rye chromosome 3R, two had 42 chromosomes each, one was a nullisomic-1R-tetrasomic-1B heterozygous for deficiency in approximately half of the long arm of chromosome 2B, and one was nullisomic-1R-trisomic-2A-trisomic-1B [65].

In this study, univalents and micronuclei were also formed in the meiosis of F_5_ hybrids, regardless of chromosome number and set completeness. Thus, genome reorganization was not completed in any of the three groups of wheat–rye F_5_ generation. Little is known about how long an allopolyploid chromosome set can remain unstable for, and how it can affect the allopolyploid evolution. Artificially resynthesized allopolyploids obtained by meiotic restitution mimic the allopolyploidization process. Studies in this field show different advances in the increase in stable allopolyploids in different taxa. An attempt to resynthesize the ancient polyploid *Arabidopsis suecica* by crossing *A. thaliana* and *A. arenosa* produced a viable hybrid, which showed homologous pairing and no important structural reorganization of the homeologous genomes in F_5_ [99]. In contrast, chromosomal variation is ubiquitous in newly developed synthetic hexaploid wheat (SHW) created by crossing *T. turgidum* with *A. tauschii* [100,101,102]. The common occurrence of univalency during meiotic metaphase I was associated with chromosome instability [102]. Young allopolyploids, termed neopolyploids, are appropriate evolutionary model systems for understanding early allopolyploid formation. Chromosome set instability is exemplified by the natural neoallotetraploids *Tragopogon mirus* and *T. miscellus* (about 40 generations). Aneuploids constitute 38 and 69% of these plants, respectively [103].

Cytological instability and aneuploidy in wheat–rye octoploid and hexaploid allopolyploids have presented problems since their creation [47,49,104,105,106]. The cytological study of triticale demonstrates that the interaction of wheat and rye genomes in the cells of one plant leads to profound derangements in cell physiology, which are maintained for decades at least. Thus, the same irregularities in meiosis and mitosis are noted in the triticale produced by Rimpau in 1889 as are found in triticale derived in later studies, including this one. In spite of the complete chromosome set, univalents are abundant in the meiosis of triticales of different ploidies [47,65,104,105,106]. In a comprehensive study of this phenomenon, only bivalents were found at diakinesis, but at MI, a pattern was established that can be interpreted as either chromosome lagging or the presence of a univalent. Aneuploid cells may arise in triticale as a result of the asynchronized functioning of rye and wheat chromosomes, and from chromosome lagging at the anaphase and telophase [105]. Chromosome disjunction depends on the proper functioning of the kinetochore [107]. As such, in stable hybrids, the CENH3 produced by one parent must be able to support the functionality of the other parent’s centromeres, despite differences in each parent’s centromere sequences [83]. Thus, the conservation of chromosome sets of the parental subgenomes in octoploid triticale over generations may be associated with the increased expression of rye centromeric histone CENH3 variants in the new genomic environment [108].

### 4.2. Rye Chromosome 4R Is Preserved until F_5_ in 1Rv(1A) × R Hybrids

Unexpectedly, we detected the preservation of rye 4R chromosomes in a monosomic or disomic state up until generation F_5_. While in F_2_, the chromosome pair 4R4R only supplemented the wheat chromosome set [69], in F_5_, 4R4R was added to wheat chromosomes in 58.59% of plants and replaced chromosomes of the fourth homeologous group in 13.15% of plants, which implies its compensational and competitive activity in a new genomic environment. The short arm of chromosome 4R is known to be homeologous to wheat chromosome arms 4BS and 4DS, and partially homeologous to chromosome arm 4AL [109], which is itself involved in evolutionary translocations between chromosome arms 4AL, 5AL, and 7BS [37,39]. On the other hand, it has been shown that rye chromosomes are incorporated into the wheat genome at different frequencies depending on cross direction and genotype [110,111]. This is true for chromosome 4R as well, which can be eliminated at high frequencies from triticale [111,112] or from disomic addition lines [113], but at the same time it can be successfully transmitted in crosses of wheat and octoploid triticale, which results in the 4R addition line [114] and in offspring from the substitution line [115]. The transmission rate of the 4R chromosome pair was consistent at 98% in subsequent generations [114]. In a study summarizing the genetic stability of several wheat–rye disomic addition lines, the frequency of progeny plants being disomic for 4R ranged from 74% to 93% [116]. In our study, the preservation of the 4R chromosome copy in F_5_ 1Rv(1A) × R likely results from the great similarity to corresponding homeologs in the genomes of wheat, similarly to the preservation of rye genes in allohexaploid triticale with a high similarity to their homeologs in *Triticum* genomes [117].

### 4.3. Alterations of Centromeric Regions

Deletions and translocations of individual chromosomal regions and chromosome arms are also among the most common chromosomal alterations [57,106]. The chromosome sets of our F_5_ hybrids contain rye and wheat telocentrics and Robertsonian translocations. The formation of inter- and intrachromosomal translocations in wheat–rye hybrids cause reductions, eliminations, or expansions in the centromeric retrotransposon sequences, and the formation of multiple centromeres [53,59]. In our experiments, a centromere carrying two nonoverlapping loci, rye-specific pAWRc and pAet06, was identified in a 1RL.1BL Robertsonian translocation. Multicentric chromosomes are frequently formed in hybrids of wheat and related species, such as *Th. elongatum, Th. poticum, Th. intermedium, Agropyron cristatum, Hordeum vulgare*, and *S. cereale* [59]. Wheat and *Th. elongatum* chromosomes with two regions containing centromeric sequences were observed in the F_1_ hybrids of null-tetra lines N3AT3B, N5BT5A, N5DT5B, and N6AT6B, and in the hexaploid amphiploid 8802 (AABBEE), which originated from hybrids between *T. durum* and *Th. elongatum* [59].

### 4.4. Meiotic Restitution Does Not Increase Ploidy in Progenitors of Octoploid Triticale

The heritability of meiotic restitution has been described in wheat hybrids [54,67,101]. The genes for meiotic restitution in those studies originated from various accessions of durum wheat *T. turgidum* [65,66,67,68]. Lines of synthetic hexaploid wheat (SHW) were produced by spontaneous chromosome doubling via unreduced gametes resulting from meiotic restitution in *T. turgidum* × *A. tauschii* hybrids [66]. These hexaploids also inherited the gene(s) for meiotic restitution, because meiotic restitution also occurs in SHW–rye F_1_ hybrids and gives rise to amphiploids or partial amphiploids [66]. Another example is line Do1, which was selected for its capacity to produce self-fertile F_1_ hybrids with rye [65,67]. Spontaneous chromosome duplication in androgenic haploids was observed when crossing the F_1_ hybrid to hexaploid triticale [67].

Three groups of F_5_ 1Rv(1A) × R hybrids were obtained via meiotic restitution, whereby chromosome pairing occurs sporadically if at all, univalents segregate into sister chromatids in meiosis I, and the second division is absent [64,118]. We found that the meiosis in some F_5_ plants of Group 3 also ended after the first division; therefore, dyads formed after division completion instead of tetrads. Another feature of chromosome behavior was the asynchronization of the meiotic cycle within an anther. Asynchronous cell division was also noted in F_5_ plants of Groups 1 and 2, but the second division did occur there. An *Arabidopsis thaliana* mutant named *tardy asynchronous meiosis* (*tam*), with a phenotype of delayed and asynchronous cell divisions during male meiosis, has been described [119]. The genes *TAM* (also known as *CYCA1;2*) and *OSD1* (*omission of second division*) are essential for the meiosis I/meiosis II transition. A mutation in *CYCA1;2/TAM* or *OSD1* leads to the premature ending of meiosis after meiosis I, and, as a consequence, to the production of diploid spores and gametes [120,121]. Hence, mutations in such genes as *OSD1* and *TAM* may favor polyploidization, as demonstrated in common wheat. A QTL responsible for the unreduction in *T. turgidum × Ae. tauschii* hybrids, named QTug.sau-3B, was identified on wheat chromosome 3B [122]. Comparative genomic analysis indicates that QTug.sau-3B is a collinear homolog of *cyca1;2/tam*, which is known to be responsible for unreduced gamete formation in *Arabidopsis thaliana* [121].

Whilst dyads in F_1_ hybrids 1Rv(1A) × R passed through mitotic divisions and formed functional pollen grains after meiosis, the mitotic division in octoploid F_5_ hybrids was greatly disturbed. The disturbances affected chromatin’s structure significantly. As a result, functional pollen grains were not formed, and plants either offered few seeds or were totally sterile. *Arabidopsis thaliana osd1* mutants showed no somatic developmental defects, male or female gametophyte lethality, or reduced fertility. Only tetraploids and triploids were found in selfed progeny [123]. In plants homozygous for null alleles of *CYCA1;2/TAM*, the unreduced gametes were functional, giving rise to polyploid progeny [121,124]. The chromosome number in each generation of *MiMe* plants’ selfing (mitosis instead of meiosis, triple *osd1/Atrec8/Atspo11-1* mutants) doubled; as such, tetraploids (4*n*, 20 chromosomes) and octoploids (8*n*, 40 chromosomes) were obtained [123]. However, the increase in ploidy was accompanied by a seed set decrease. Fertility dropped from 25 ± 6 seeds/fruit in 2*n* plants and 19 ± 4 in 4n plants to < 0.1 in 8*n* plants. The causes of this phenomenon remain obscure. In the case of octoploid plants 1Rv(1A) × R, the formation of microspores with 56 chromosomes may induce stress, entailing a collapse in cell cycle regulation and, as a consequence, apoptosis [125]. The Poaceae family includes perennial plants with over 100 chromosomes [126]. They are characterized by low fertility and sterility, probably associated with meiotic anomalies induced by polyploidy. Studies of the genus *Arundo* L. (Poaceae) have demonstrated that the sterility of *A. micrantha* (2*n* = 12x) and *A. donax* (2*n* = 18x) is due to the early failure of gametogenesis [127]. In theory, unreduced gametes form during meiosis in these species; however, these gametes have not been proven to cause sterility.

Is there a limit to ploidy in flowering plants? Although the haploid chromosome numbers in 66120 angiosperm species with known chromosome sets vary from *n* = 2 to *n* = 320 [126], the chromosome numbers of 80% of angiosperms range from *n* = 5 to 20, and in 95% the haploid chromosome numbers are less than *n* = 34. A similar distribution in chromosome numbers is seen in the tribe Triticeae. All the species of this tribe have the basic haploid chromosome number *x* = 7. About 31% of the species are diploids (or rather paleopolyploids); 1% are triploids; 45%, tetraploids; 17%, hexaploids; 5%, octoploids; 0.2%, decaploids; and 0.2%, dodecaploids. *Elymus* displays the larger series and highest level of polyploidy, from 2*x* to 12*x* [20]. Owing to the cyclic mode of polyploidy, most angiosperm species have less than 14 chromosome pairs, which show no signs of exponential growth [128]. Genome synteny comparisons show that many ancient polyploidization events were followed by striking reductions in chromosome number [16], which in some cases are estimated to have occurred relatively soon after polyploidization [129]. For instance, an *n* = 7 monocot ancestor underwent four tetraploidy events in the lineage leading to *Zea mays*; had not it been for fusions, maize would have *n* = 112, but today it has *n* = 10 [130]. On the other hand, chromosome sizes cannot rise infinitely after fusion. Chromosome lengths are limited by the sizes of the dividing cells; chromosome arms longer than half the cell length are truncated by the new cell wall, causing damage and gene loss at the ends [131], as proven experimentally with artificial chromosomes in barley [132]. Ploidy increase may also be limited by other factors as well [133]. These include biochemical and energetical expenses, cell size limits, time limitations caused by longer mitosis and meiosis with larger genomes, and difficulties in support of gene expression diversity in giant genomes in response to environmental changes.

## 5. Conclusions

In this work, we studied the karyotypes and meiotic behavior of chromosomes in three groups of fifth-generation hybrids (1Rv(1A) × R) obtained via meiotic restitution. Our observations suggest that genome reorganization is not finished in any of the groups of F_5_ hybrids. It was found that in two groups of karyotypes, one to three rye chromosomes were preserved in a disomic or monosomic state. The chromosome 4R in 13.15% of plants substituted the chromosomes of the fourth group of wheat genomes, ABD. The karyotypes of the plants of these groups were also characterized by the presence of Robertsonian translocations. The chromosome sets of Group 3 were near octoploid, varying from 52 to 56. The presence of 16 rye chromosomes owing to chromosome 1R tetrasomy (68.4% of plants) was a distinctive feature.

Meiosis in the hybrids was unstable. Univalents in the first division were found, characterized by a violation of segregation, which led to the formation of micronuclei in microspores. However, according to the correlation analysis, no connection was found between the presence of micronuclei and seed sets. The analysis of meiosis in Group 3 revealed asynchronous cell division and omission of the second division. Diploid gametes did not form because of significant disturbances during mitosis in gametogenesis. As a result, the frequency of the formed dyads was negatively correlated (r = −0.73) with the seed sets. Thus, the trait “meiotic restitution” is inherited in octoploid triticale; however, gametogenesis does not take place in dyads, and functional gametes are not formed.

## Figures and Tables

**Table 1:** Material analyzed.

Hybrid F_2_	Hybrid F_3_	Hybrid F_4_	Hybrids F_5_					
			Routine Analysis			FISH		
			Plants	Anthers	Meiocytes+ Microspores	Plants	Anthers	Meiocytes
6-1 (subgroup *1a*)	22-4	72-3	7	15	1419	3	7	228
		72-4	4	11	826	5	10	489
		72-11	4	17	1851	2	7	315
		72-12	6	23	2934	6	10	684
6-2 (subgroup *1b*)	23-8	76-1	11	27	3304	-	-	-
	23-10	77-1	4	11	1007	-	-	-
		77-4	6	16	1482	-	-	-
		77-8	6	12	961	-	-	-
73-1 (group 3)	41-7	87-1	9	43	3215	3	7	112
	41-16	88-1	9	47	2862	2	5	72
		88-2	6	34	3117	-	-	-
	41-22	89-6	8	41	3096	3	6	82

**Table 2:** Criteria for interpreting the strength of the relationship between two variables.

Correlation Coefficient	Interpretation
0.90 to 1.00 (−0.90 to −1.00)	Very high positive (negative) correlations, very dependable associations
0.70 to 0.90 (−0.70 to −0.90)	High positive (negative) correlations, marked associations
0.50 to 0.70 (−0.50 to −0.70)	Moderate positive (negative) correlations, substantial associations
0.30 to 0.50 (−0.30 to −0.50)	Low positive (negative) correlations, defined but small associations
0 to 0.30 (0 to −0.30)	Negligible correlations

**Table 3:** Combinations of rye and wheat chromosomes of the 4th homeologous group.

Homeologous Group 4	Plants/%
AABBDDRR	76/58.9%
A-BBDDRR	1/0.77%
AA-BDDRR	4/3.1%
AABB-DRR	3/2.32%
--BBDDRR	1/0.77%
AA--DDRR	5/3.87%
A-B-DDRR	1/0.77%
A-BB-DRR	2/1.55%
AABBDD-R	3/2.32%
AA-BDDR-	1 /0.77%
AABBDD-RL	2/1.55%
AABBD-RRL	1/0.77%
AABBLDDRR	1/0.77%
AABLBLDDRR	7/5.43%
AABBDDRS-	1/0.77%
AABBDDRRR	1/0.77%
AABBDD--	19/14.73%
Total	129/100%

**Table 4:** Micronucleus formation at telophase II and seed sets in F_5_ plants of Group 1.

Hybrid F_1_	Hybrid F_2_	Hybrid F_3_	Hybrid F_4_	Hybrids F_5_	
				Meiocytes with Micronuclei, %	Seed Set
**4-7**	6-1 (subgroup *1a*)	22-4	72-3	47 ± 3.75	67 ± 6.2
			72-4	56.35 ± 8.3	61.83 ± 9.2
			72-11	70.26 ± 6.2	81.88 ± 9.4
			72-12	60.37 ± 6.0	102.47 ± 9.3
	6-2 (subgroup *1b*)	23-8	76-1	13.93 ± 1.89	55.93 ± 6.7
		23-10	77-1	65.21 ± 9.4	37.19 ± 8.5
			77-4	46.5 ± 2.45	40.92 ± 7.9
			77-8	46.95 ± 7.95	40 ± 6.2

## Data Availability

Data are contained within the article or supplementary material.

## Supplementary Materials

The following are available online at https://www.mdpi.com/article/10.3390/plants10102052/s1, Table S1: Origin of F_5_ wheat–rye hybrids and number of karyotype studied.
