# Supplementary material for: Karyotype Reorganization in Wheat–Rye Hybrids Obtained via Unreduced Gametes: Is There a Limit to the Chromosome Number in Triticale?
Source: Plants (Basel). 2021 Sep 29;10(10):2052. doi: 10.3390/plants10102052 (PMC8538156; doi:10.3390/plants10102052)
Supplement: Supplementary file 1 [file plants-10-02052-s001.zip › plants-1356285-supplementary.pdf]

Table S1

Origin of F_5 wheat-rye hybrids and number of karyotype studied

Hybrid F_2	Hybrid F_3	Hybrid F_4	F_5 hybrids karyotypes, FISH	F_5 hybrids karyotypes, C-banding
6-1 (6 kernels) (subgroup 1a)	22-4 (32 kernels)	72-3 (146 kernels)	9	7
		72-4 (139 kernels)	9	7
		72-11 (103 kernels)	10	7
		72-12 (112 kernels)	12	7
6-2 (20 kernels) (subgroup 1b)	23-8 (1 kernel)	76-1 (154 kernels)	10	6
	23-10 (15 kernels)	77-1 (104 kernels)	15	4
		77-4 (77 kernels)	9	4
		77-8 (137 kernels)	13	3
	23-13 (45 kernels)	109-1 (145 kernels)	5	4
		109-16 (117 kernels)	2	4
		109-17 (180 kernels)	2	6
		109-20 (245 kernels)	1	5
		109-26 (193 kernels)	3	5
		109-28 (156 kernels)	2	6
		109-31 (105 kernels	2	6
		109-34 (207 kernels)	2	5
7-3 (126 kernels) (group 2)	24-2 (39 kernels)	44-8 (84 kernels)	-	6
		44-11 (48 kernels)	-	4
	24-9 (36 kernels)	45-7 (49 kernels)	-	3
		45-14 (75 kernels)	-	5
	24-12 (28 kernels)	46-2 (101 kernels)	-	4
		46-13 (68 kernels)	-	2
	24-14 (21 kernels)	47-4 (88 kernels)	-	2
		47-10 (66 kernels)	-	4
	24-16 (30 kernels)	48-5 (66 kernels)	-	3
		48-11 (66 kernels)	-	4
7-4 (320 kernels) (group 2)	28-8 (73 kernels)	49-7 (62 kernels)	-	3
		49-9 (55 kernels)	-	2
		49-12 (61 kernels)	-	4
	28-11 (162 kernels)	50-11 (82 kernels)	-	5
	28-20 (107 kernels)	51-4 (96 kernels)	-	2
		51-9 (109 kernels)	-	6
	28-21 (142 kernels)	52-5 (100 kernels)	-	4
		52-7 (106 kernels)	-	7
		52-13 (96 kernels)	-	4
	28-24 (134 kernels)	53-9 (100 kernels)	-	1
		53-11 (139 kernels)	-	3
7-5 (67 kernels) (group 2)	31-13 (100 kernels)	54-6 (80 kernels)	-	4
		54-9 (73 kernels)	-	5
		54-11 (117 kernels)	-	5
	31-17 (165 kernels)	55-3 (122 kernels)	-	5
		55-10 (108 kernels)	-	3
		55-15 (70 kernels)	-	4
	31-22 (165 kernels)	56-5 (85 kernels)	-	4
		56-8 (97 kernels)	-	3

		56-13 (103 kernels)	-	2
	31-23 (133 kernels)	57-5 (88 kernels)	-	4
		57-7 (99 kernels)	-	4
		57-8 (128 kernels)	-	3
	31-28 (128 kernels)	58-3 (136 kernels)	-	4
		58-5 (104 kernels)	-	4
		58-9 (79 kernels)	-	4
26-1 (35 kernels) (group 3)	41-7 (11 kernels)	87-1 (75 kernels)	9	6
	41-16 (12 kernels)	88-1 (55 kernels)	8	5
		88-2 (54 kernels)	9	5
	41-22 (14 kernels)	89-6 (117 kernels)	11	4
